# Valorization of Whey into a Bioactive Hydrolysate with ACE-Inhibitory Activity and Food Application Potential

**DOI:** 10.3390/foods15142516

**Published:** 2026-07-16

**Authors:** María Angélica Fellenberg, Wladimir Silva-Vera, Olga Panes, Romina L. Abarca

**Affiliations:** 1 Departamento de Ciencias Animales, Facultad de Agronomía y Sistemas Naturales, Pontificia Universidad Católica de Chile, Macul, Santiago 7820436, Chile; mafellen@uc.cl (M.A.F.); opanes@uc.cl (O.P.); 2 Departamento de Biotecnología, Universidad Tecnológica Metropolitana, Las Palmeras 3360, Ñuñoa 7800003, Chile; w.silvav@utem.cl

**Keywords:** whey protein hydrolysate, ACE inhibition, peptide profile, processing stability, dairy products, functional beverages

## Abstract

Bioactive peptides derived from whey proteins have attracted increasing interest as natural dietary modulators of angiotensin-converting enzyme (ACE) activity and potential contributors to cardiovascular health. In this study, bioactive whey hydrolysate (BWH) was obtained by controlled enzymatic hydrolysis with plant-derived proteases and characterized in terms of physicochemical properties, peptide profile, ACE-inhibitory activity, stability, and performance in food matrices. RP-HPLC and LC–MS/MS analysis revealed a complex, reproducible peptidome across production batches, including a conserved set of antihypertensive peptide sequences. BWH exhibited high ACE-inhibitory activity (>90%) with low intra-batch variability, and dose–response analysis yielded an estimated IC_50_ of 3.20% BWH under the assay conditions. Its bioactivity and peptide profile remained stable during storage at room temperature and under refrigeration, as well as after mild thermal treatment (pasteurization), although partial activity loss was observed after baking at high temperatures. Incorporation trials showed that BWH retained strong ACE-inhibitory functionality when incorporated at 7% into yogurt and orange juice, with minimal effects of pasteurization in the beverage matrix. These findings support the potential use of BWH as a functional ingredient for developing antihypertensive foods and beverages.

## 1. Introduction

Cardiovascular disease (CVD) remains the leading cause of mortality worldwide, with hypertension as one of its most prevalent and modifiable risk factors. Hypertension is commonly defined as systolic blood pressure (SBP) ≥ 140 mmHg and/or diastolic blood pressure (DBP) ≥ 90 mmHg, based on repeated measurements [1]. The global prevalence of hypertension surpasses 1.4 billion adults aged 30–79 years, representing 33% of the population in this age range, and projections indicate a continuous rise associated with aging populations, dietary changes, and sedentary lifestyles [2,3,4,5]. Although pharmacological therapies such as diuretics, beta blockers, alpha blockers, neprilysin inhibitors [6], angiotensin-converting enzyme (ACE) inhibitors, angiotensin receptor blockers (ARBs), and calcium channel blockers effectively reduce cardiovascular risk, their long-term use may lead to side effects and poor adherence [7,8]. Consequently, increasing attention has been directed toward natural and food-based strategies capable of modulating blood pressure through physiological pathways [9,10,11,12].

Dietary strategies such as the Mediterranean and DASH dietary patterns have been associated with antihypertensive effects due to their contribution of fiber, antioxidants, minerals, unsaturated fatty acids, and other cardioprotective compounds [13]. In addition, food-derived bioactive peptides have gained attention as dietary components capable of modulating blood pressure through mechanisms such as endothelial protection, attenuation of oxidative stress, and ACE inhibition [14].

Bioactive peptides (BPs) are defined as short amino acid sequences (2–20 residues) that exert biological functions beyond basic nutrition. These peptides display antioxidant, immunomodulatory, antimicrobial, and, notably, antihypertensive activity through ACE inhibition [15,16,17]. Within the diverse sources of bioactive peptides, dairy proteins, particularly whey proteins (WPs), stand out for their exceptional digestibility, amino acid balance, and demonstrated health benefits [18,19,20,21,22,23]. Recent reviews have emphasized that whey proteins are among the most promising precursors of bioactive peptides because enzymatic hydrolysis can release sequences with ACE-inhibitory, antioxidant, immunomodulatory, and metabolic effects. However, the biological relevance of these peptides depends not only on their release from the parent proteins but also on their stability, peptide profile, processing tolerance, and performance in food systems. Therefore, the development of whey-derived hydrolysates as functional ingredients requires an integrated evaluation that goes beyond the initial demonstration of ACE-inhibitory activity [21,24].

Whey is the principal by-product of the cheese-making process and represents a major underutilized biomass in the dairy sector. Approximately 9 L of whey are generated for every 10 L of milk processed into cheese, and this by-product retains a substantial fraction of the original milk nutrients, including soluble proteins, peptides, lactose, and minerals [25,26,27,28,29]. The large volume of whey generated by cheese production makes it an abundant and inexpensive raw material for developing functional ingredients and supporting circular-economy initiatives within the dairy industry.

Sweet whey was selected in this study because it is one of the main by-products generated during cheese manufacture and contains soluble proteins, particularly β-lactoglobulin and α-lactalbumin, which are recognized precursors of ACE-inhibitory peptides. Compared with acid whey, sweet whey generally has a less acidic composition, which may favor enzymatic hydrolysis, peptide stability, and subsequent incorporation into food matrices. Thus, sweet whey represents a suitable raw material for obtaining peptide-rich hydrolysates while contributing to the valorization of dairy by-products [24,27,30].

Historically, whey disposal posed environmental challenges due to its high biological oxygen demand (BOD); however, advances in biotechnology have enabled its valorization into bioactive hydrolysates and peptide-rich functional products [29,30,31,32,33]. Controlled enzymatic hydrolysis of whey proteins, mainly β-lactoglobulin and α-lactalbumin, releases encrypted peptide sequences with various physiological functions, including ACE inhibition, which is particularly relevant for cardiovascular health [34,35].

Enzymatic hydrolysis is one of the most widely used strategies for releasing encrypted bioactive peptides from food proteins. Although microbial and animal proteases have been extensively used for whey protein hydrolysis, plant-derived proteases have gained increasing attention because of their food-grade applicability, broad catalytic specificity, and ability to generate diverse peptide profiles. Proteases such as papain, bromelain, ficin, and other plant cysteine proteases have been reported to release low-molecular-weight peptides with antioxidant, antihypertensive, and other bioactive properties. In this context, plant-derived proteases may represent an alternative hydrolysis strategy for generating multifunctional whey-derived peptide fractions [34,36].

The renin–angiotensin system (RAS) plays a crucial role in blood pressure regulation. ACE catalyzes the conversion of angiotensin I into angiotensin II, a potent vasoconstrictor that increases vascular resistance. Excessive ACE activity has been associated with hypertension, endothelial dysfunction, and chronic cardiovascular disorders [37,38]. Accordingly, natural dietary ACE inhibitors, particularly those derived from whey proteins, represent promising alternatives or adjuncts to conventional synthetic antihypertensive drugs, offering the potential to modulate blood pressure through milder and safer physiological mechanisms [39,40].

ACE-inhibitory peptides from whey hydrolysates, such as Val-Pro-Pro (VPP) and Ile-Pro-Pro (IPP), have shown significant blood pressure-lowering effects in both animal and human models [41]. Recent advances in peptidomics and in silico screening have revealed thousands of additional sequences with potential antihypertensive activity, often characterized by short chain length (<12 amino acids) and hydrophobic or aromatic residues at the C-terminal region, showing an enhanced ACE-binding affinity [42,43,44]. Thus, beyond ACE inhibition, whey-derived peptides have also exhibited antioxidant, immunomodulatory, and gut-microbiota-modulating activities, expanding their potential applications as functional food ingredients [8,24,45,46,47]. Nevertheless, the identification of potentially bioactive sequences alone is insufficient to support food application, since peptide release and activity may vary according to raw material composition, enzyme specificity, hydrolysis conditions, drying technology, storage, and interactions with food matrix components [48].

Several challenges remain in translating peptide bioactivity into viable food systems. Processing technologies and storage conditions such as heating, pH variation, dehydration, or long-term storage may cause peptide degradation or conformational changes that diminish bioactivity through Maillard-type reactions [49,50,51]. Moreover, peptide stability and bioavailability in vivo are limited by gastrointestinal hydrolysis and poor intestinal absorption [52]. To address these issues, recent research has explored non-thermal processing technologies and encapsulation systems, such as high-pressure processing (HPP), ultrasonication, microwave-assisted hydrolysis, and nano- or micro-encapsulation, which have shown promise in preserving ACE-inhibitory activity and improving peptide delivery [53].

Although whey-derived ACE-inhibitory peptides have been widely investigated, important gaps remain in their translation into functional food ingredients. Many studies have focused mainly on peptide release, ACE-inhibitory activity, or in silico identification of active sequences, whereas fewer have integrated industrial whey valorization, plant-protease hydrolysis, batch-to-batch peptide reproducibility, physicochemical characterization of the dried hydrolysate, processing stability, and functionality after incorporation into real food matrices. This integrated validation is essential because peptide bioactivity may be affected by hydrolysis conditions, drying, storage, heat treatments, and interactions with food components. Therefore, the present study aimed to produce a bioactive whey hydrolysate (BWH) from sweet whey using plant-derived proteases and to characterize its physicochemical properties, peptide profile, ACE-inhibitory potency, storage and thermal stability, and performance after incorporation into yogurt and orange juice. This approach provides a broader technological and functional assessment of BWH as a potential ingredient for the development of foods with antihypertensive potential.

## 2. Materials and Methods

Sweet whey of bovine origin (*Bos taurus*) was obtained from a Chilean dairy company dedicated to the production of ripened cheese and used as raw material for the production of the bioactive whey hydrolysate (BWH). Food-grade plant-derived proteases supplied by Enzyme Development Corporation (Scranton, PA, USA) were used for enzymatic hydrolysis. Angiotensin-converting enzyme (ACE; EC 3.4.15.1; product code A6778) was purchased from Sigma-Aldrich (St. Louis, MO, USA). Hippuryl-histidyl-leucine (HHL), acetonitrile, trifluoroacetic acid (TFA), and all other reagents were HPLC -grade reagents were purchased from Merck KGaA (Darmstadt, Germany). ACE-inhibitory activity was determined spectrophotometrically at 228 nm using a UV-1650PC spectrophotometer (Shimadzu Corporation, Kyoto, Japan). pH was measured using a Touch PH-P pH meter (BEL Engineering s.r.l., Monza, Italy). Moisture content was determined using an SDT Q600 thermogravimetric analyzer (TA Instruments, New Castle, DE, USA). Water activity was measured using a BWA-4B water activity meter (Biobase, Biodustry (Shandong) Co., Ltd., Jinan, China), and color parameters were determined using a CR-410 colorimeter (Konica Minolta, Inc., Tokyo, Japan). Peptide profiling was performed by reverse-phase HPLC using a modular Prominence HPLC system with UV detection (Shimadzu, Corporation, Kyoto, Japan) equipped with a C18 column, (5 µm, 150 mm × 2.1 mm; column supplier information was not available in the archived chromatographic record). Peptide identification was carried out using a nanoElute nanoUHPLC system coupled to a timsTOF Pro high-resolution mass spectrometer (Bruker Daltonics GmbH & Co. KG, Bremen, Germany). Spray drying was conducted at laboratory scale using an X-22870 spray dryer (Lab-Plant Ltd., Huddersfield, UK), and freeze-drying was performed at laboratory scale using a FreeZone 8 L freeze dryer (Labconco, Corporation, Kansas City, MO, USA).

### 2.1. Production of Bioactive Whey Hydrolysate (BWH)

Hydrolysis was performed at pH 6.5–7.0 and 50–55 °C under continuous stirring for 1 h using two food-grade plant-derived protease preparations supplied by Enzyme Development Corporation (Scranton, PA, USA). The enzyme preparations were added at enzyme-to-substrate ratios of 3.97 U/g protein and 1.55 U/g protein, respectively.

After hydrolysis, the enzymatic reaction was terminated by heating the hydrolysate to 85 °C for 10 min to inactivate the proteases. The hydrolyzed whey was clarified by centrifugation at 10,000× *g* for 20 min at 4 °C to remove insoluble material. At an initial stage, two drying methodologies were evaluated to obtain BWH in powder form: spray drying and freeze-drying. Differences between the two drying methods were assessed based on physicochemical parameters.

For spray drying, the clarified supernatant was dried using a laboratory-scale spray dryer (Lab-Plant Ltd., Huddersfield, UK) under the following conditions: inlet air temperature 170 °C, outlet air temperature 85 °C, feed flow rate 5 mL·min^−1^, and two-fluid nozzle atomization.

For freeze-drying, the clarified hydrolysate was frozen at −40 °C and subsequently dried in a pilot-scale freeze dryer under vacuum conditions (≤0.1 mbar), with a condenser temperature of approximately −50 °C, until constant mass was achieved.

The BWH samples obtained by freeze-drying and spray drying were physicochemically characterized and compared. Based on these analyses, with particular emphasis on water activity and considering the industrial relevance of spray drying for the production of powdered dairy ingredients, the most suitable drying method was selected for subsequent analyses and studies, taking into account its potential for future scale-up to industrial plants processing high whey volumes.

### 2.2. Physicochemical Characterization of BWH

#### 2.2.1. pH Measurement

The pH of whey samples and peptide-containing fractions was determined using a calibrated Touch PH-P pH meter with a resolution of 0.001 pH units (BEL Engineering s.r.l., Monza, Italy). Prior to measurements, the instrument was calibrated at room temperature using standard buffer solutions (pH 4.00, 7.00, and 10.00) according to the manufacturer’s instructions. Samples were gently homogenized to ensure uniformity and measured at 25 °C. The electrode was rinsed with deionized water and gently dried between measurements to avoid cross-contamination. All pH determinations were performed in triplicate.

#### 2.2.2. Apparent Density Determination

The apparent density (ρ_ap._) of the whey-derived peptide powder was determined following gravimetric principles consistent with ISO 3923-1 [54] (Metallic powders—Determination of apparent density) and ASTM D7481 [55] (Standard Test Method for Bulk Density of Powders), adapted for food-grade materials. A clean, dry container of known volume (V) was filled with the powder without compaction, allowing the material to settle under gravity. Excess powder was carefully leveled using a straight edge to ensure a constant volume. The filled container was then weighed using an analytical balance, and the mass of the powder (m_i_) was obtained by difference. Apparent density (ρ_ap_) was calculated as the ratio of powder mass to container volume. Measurements were performed in triplicate.

#### 2.2.3. Moisture Content Measurement by Thermogravimetric Analysis

The sample moisture content was determined using a thermogravimetric moisture analyzer (TA Instruments, New Castle, DE, USA), based on continuous mass loss during controlled heating. Prior to analysis, the instrument was warmed up and verified according to the manufacturer’s recommendations. Approximately 1.5 g of powder sample was evenly spread as a thin layer on the analyzer pan. Samples were dried at 130 °C under halogen heating for 15 min. Moisture content was calculated gravimetrically, and all measurements were performed in triplicate.

#### 2.2.4. Water Activity Determination

Water activity (a_w_) was measured using a water activity meter (Biobase, Biodustry (Shandong) Co., Ltd., Jinan, China). Approximately 1.5 g of sample was placed in a clean, dry sample cup, ensuring that the material covered the base without compacting. Measurements were conducted at 25.4 ± 0.5 °C, and equilibrium was considered reached when the instrument stability criterion was satisfied. All determinations were performed in triplicate.

#### 2.2.5. Chromatic Parameters

The chromatic parameters a*, b*, and L* were measured following the methodology described by Silva-Vera, W. et al. (2025) [56]. Briefly, the color values a* (−greenness/+redness), b* (−blueness/+yellowness), and L* −black/+white) of the samples were determined using a portable digital colorimeter (CR-400 Series, V.1.13, Konica Minolta, Inc., Tokyo, Japan) on random spots. A reference white calibration plate was employed for standardization (L* = 94.65, a* = −0.84, b* = 4.5). The chromatic parameters (CieLab) and whiteness index (WI) of powder samples were calculated using the respective equations:

(1) $$WI\;=\;100\;-\;\sqrt{{\left(100\;-{\;L}^{*}\right)}^{2}\;+\;a^{*2}\;+{\;b}^{*2}}$$

where L* represents lightness, a* represents the transition from green to red color, and b* represents the transition from blue to yellow.

### 2.3. Peptide Characterization

#### 2.3.1. Peptide Profile by HPLC

Peptide profiles of BWH were analyzed by high-performance liquid chromatography (HPLC) using a reverse-phase C18 column. Samples of BWH obtained from different industrial whey batches were evaluated to assess potential variability among production lots. Separation was achieved using a gradient elution with 0.1% (*v*/*v*) trifluoroacetic acid in water and 0.1% (*v*/*v*) trifluoroacetic acid in acetonitrile as mobile phases. Elution was monitored at 214 nm, with a total run time of 60 min, a flow rate of 0.75 mL·min^−1^, and the column temperature set at 40 °C. This methodology was based on the protocol described by Zheng et al. (2022), with minor modifications [44].

#### 2.3.2. Peptide Sequencing by LC–MS/MS

Peptide identification and sequencing were performed by liquid chromatography coupled to tandem mass spectrometry (LC–MS/MS). Prior to analysis, samples were reduced, alkylated, desalted using C18 spin columns, and concentrated. Peptides were analyzed using a nanoUHPLC system coupled to a high-resolution mass spectrometer operating in positive electrospray ionization mode. Data acquisition was performed using data-dependent acquisition, and peptide identification was conducted against the *Bos taurus* proteome database using appropriate bioinformatics software. LC–MS/MS data were analyzed using PEAKS Studio X+ (Bioinformatics Solutions Inc., Waterloo, ON, Canada) for *de novo* peptide identification. The analysis was performed using a no-enzyme/non-specific cleavage approach, with monoisotopic masses, a precursor mass tolerance of 50 ppm, and a fragment ion tolerance of 0.05 Da. Peptide sequences were filtered using an Average Local Confidence (ALC) score > 80%. Peptide origin was assessed against the *Bos taurus* UniProt proteome database, (UniProt Consortium; https://www.uniprot.org/; accessed on 14 December 2025),and antihypertensive peptide annotation was performed using a customized database of previously reported antihypertensive peptides.

### 2.4. Angiotensin-Converting Enzyme (ACE)-Inhibitory Activity

Angiotensin-converting enzyme (ACE)-inhibitory activity of three bioactive whey hydrolysate (BWH) samples obtained from different production batches (BWH 1–BWH 3) was determined using a spectrophotometric assay based on the hydrolysis of hippuryl-histidyl-leucine (HHL) as substrate. BWH samples were initially dissolved in distilled water to obtain a stock solution of 170 mg/mL. For the assay, 50 µL of this solution was mixed with 200 µL of hippuryl–borate saline buffer (0.1 M sodium borate, 0.3 M NaCl, pH 8.3) containing 5 mM HHL. The enzymatic reaction was initiated by adding 20 µL of angiotensin-converting enzyme (ACE) solution (0.2 IU/mL). The reaction mixture was incubated for 40 min at 37 °C. The reaction was terminated by the addition of 250 µL of 1 M HCl. The hippuric acid released during the reaction was extracted and quantified by measuring absorbance at 228 nm using a spectrophotometer. ACE-inhibitory activity was expressed as percentage inhibition relative to a control reaction performed in the absence of BWH. Each BWH sample was analyzed in triplicate (*n* = 3), and results were expressed as mean ± standard deviation. Statistical analysis was performed using one-way analysis of variance (ANOVA) followed by Tukey’s honestly significant difference (HSD) post hoc test. Differences were considered statistically significant at *p* < 0.05. The experimental procedure was adapted from the methodology reported by Mansinhbhai et al. (2023) [45].

ACE-inhibitory activity was expressed as percentage inhibition relative to a control reaction performed in the absence of BWH. To further evaluate the inhibitory potency of BWH, a dose–response assay was performed using different BWH concentrations. The IC_50_ value, defined as the BWH concentration required to inhibit 50% of ACE activity, was estimated from the fitted inhibition curve. Captopril, a pharmacological ACE inhibitor, was used as a positive control to verify the responsiveness of the assay. Blank corrections were applied when appropriate to account for background absorbance from reagents and samples. Each BWH sample was analyzed in triplicate (*n* = 3), and results were expressed as mean ± standard deviation.

### 2.5. Stability Studies

In order to evaluate the functional and structural stability of bioactive whey hydrolysate (BWH) under processing and storage conditions relevant to food applications, the ingredient was reconstituted in distilled water at 17% (*w*/*v*), a concentration representative of a potential technological dosage for incorporation into food matrices. The reconstituted samples were subjected to controlled storage conditions and thermal treatments simulating common industrial and domestic scenarios.

For storage stability assessment, reconstituted BWH samples were placed in sealed containers and stored at room temperature (20–25 °C) for 30 days and under refrigeration (4–8 °C) for 68 days. Samples were maintained protected from direct light during storage. At predetermined sampling times, aliquots were withdrawn for the determination of angiotensin-converting enzyme (ACE)-inhibitory activity and peptide profile analysis by high-performance liquid chromatography (HPLC).

Thermal stability was further evaluated by subjecting the BWH samples to two processing conditions: slow pasteurization (62–64 °C for 30 min) and a baking-like condition (170 °C for 30 min). After thermal treatment, samples were cooled to room temperature prior to analysis.

ACE inhibitory activity was determined using a spectrophotometric assay based on the hydrolysis of hippuryl-histidyl-leucine (HHL), and results were expressed as percentage inhibition. The peptide profile of BWH before and after storage and thermal treatments was analyzed by reverse-phase HPLC using a C18 column with UV detection. Chromatograms were qualitatively compared to assess potential changes in the global peptide distribution.

### 2.6. Incorporation of BWH into Food Matrices

Preliminary incorporation trials of bioactive whey hydrolysate (BWH) were conducted in selected food matrices to evaluate technological feasibility and the initial stability of its bioactivity within real food systems. Two representative matrices were considered: a fermented dairy product (yogurt) and fruit-based beverages (orange juice). Each experimental condition was analyzed in triplicate. After incorporation and during storage, samples were analyzed for angiotensin-converting enzyme (ACE) inhibitory activity and peptide profile. Data were subjected to one-way analysis of variance (ANOVA), and mean comparisons were performed using Tukey’s post hoc test, with statistical significance set at *p* < 0.05.

The BWH addition level of 7% was selected as a preliminary incorporation level to evaluate whether the hydrolysate could retain ACE-inhibitory activity after addition to representative food matrices, rather than as an optimized final dose. This concentration was considered technologically feasible for incorporation into both yogurt and orange juice and sufficiently high to allow measurable ACE-inhibitory activity despite possible interactions with matrix components. Further formulation, sensory, and dose-optimization studies will be required to define the optimal incorporation level for specific food applications.

#### 2.6.1. Developed Yogurt

A control yogurt was prepared at laboratory scale without additives, using only whole milk (3.1% fat) and commercial starter cultures. In parallel, a commercial yogurt sample was analyzed for comparative purposes. Additionally, a laboratory-produced yogurt fortified with 7% (*w*/*w*) BWH was prepared. For yogurt production, whole milk was heated to 43–45 °C prior to inoculation with the starter culture, and fermentation was carried out at 43–45 °C until a final pH of 4.5–4.8 was reached, with a minimum fermentation time of 4 h. After fermentation, yogurt samples were sealed and stored under refrigerated conditions (4–8 °C) until analysis.

#### 2.6.2. Developed Juice

Orange juice samples included freshly squeezed natural orange juice prepared at laboratory scale, a commercially available orange juice, and freshly squeezed natural orange juice fortified with 7% (*w*/*v*) BWH. After preparation and fortification, samples were stored under refrigerated conditions (4–8 °C) and at room temperature (20–25 °C) to simulate different storage conditions. Samples were collected during storage for subsequent determination of ACE-inhibitory activity and peptide profile.

### 2.7. Statistical Analysis

Statistical analysis was performed using STATGRAPHICS Centurion 19 software (Statgraphics Technologies, Inc., The Plains, VA, USA) Results were expressed as mean ± standard deviation. Analyses were conducted using three independent samples or production batches, and each analytical determination was performed in triplicate. Normality and homogeneity of variance were evaluated prior to statistical comparison. When these assumptions were met, data were analyzed by one-way analysis of variance (ANOVA), followed by Tukey’s HSD test for multiple comparisons. Statistical significance was established at *p* < 0.05. For experiments involving more than one factor, two-way ANOVA was considered; however, one-way ANOVA was applied when comparisons corresponded to predefined independent treatment groups rather than complete factorial designs.

## 3. Results and Discussion

### 3.1. Physicochemical Evaluation of the BWH

Table 1 presents the physicochemical parameters evaluated in bioactive whey hydrolysate (BWH) obtained by freeze-drying and spray-drying.

### 3.2. pH Measurements

The spray-dried BWH sample exhibited a significantly higher pH value (7.5 ± 0.2) compared to the lyophilized samples, which remained close to neutrality (6.7–6.8) (*p* ≤ 0.05) (Table 1). Although statistically different, the pH values of all powders were confined to a narrow neutral-to-mildly alkaline range, indicating that the drying approach induced only moderate shifts in acid–base balance. In peptide-based systems, pH is a relevant technological parameter influencing solubility, electrostatic interactions, and stability in aqueous environments. The slightly higher pH observed after spray drying may reflect processing-related concentration effects or subtle changes in mineral equilibrium rather than chemical degradation, since no extreme alkaline conditions were reached. Similar behavior has been described for whey protein hydrolysates, where near-neutral pH conditions support peptide dispersion and functional performance in food matrices [57].

### 3.3. Apparent Density

The apparent density of the powders provides insight into their physical structure and porosity. BWH Lyophilized Prev. Crist-Filtr. exhibits a significantly lower density (0.31 g/mL) compared to BWH (Lyophilized) (0.59 g/mL) and BWH (Spray dried) (0.62 g/mL). This highlights a fundamental structural difference between the samples. Lyophilization typically produces a highly porous, “glass-shard” or laminar structure caused by the sublimation of ice crystals, which leaves behind voids where the ice previously resided. The significantly lower density in the pre-treated sample (BWH Lyophilized) suggests that the crystallization and filtration steps facilitated a more rigid and porous framework. Removal of solids through filtration and the organization of lactose into crystals before freezing likely resulted in a different ice crystal matrix, leading to a lighter powder after sublimation. In contrast, the higher density in the spray-dried sample (BWH Spray dried) is characteristic of the spherical, non-porous, and often hollow particles produced by atomization in hot air, which tend to pack more tightly [58]. Therefore, spray drying was selected for subsequent analyses, as it provided more favorable technological characteristics and represents a more scalable and cost-effective alternative than freeze-drying.

### 3.4. Water Activity

Despite the significant differences in total moisture content, the water activity (a_w_) showed no statistically significant differences among the three samples (*p* > 0.05), with values ranging from 0.29 to 0.36. Water activity is a more reliable predictor of stability than total moisture, as it represents the energy state of the water and its availability for chemical and microbial reactions. All three samples fall within the range of “safe” a_w_ values for powdered products, where the risk of microbial growth is eliminated, and the rate of degradative reactions like lipid oxidation and non-enzymatic browning is minimized [59].

### 3.5. Chromatic Analysis and Whiteness Index

The spray-dried sample is significantly lighter (L* = 97.96) and possesses a much higher Whiteness Index (92.18) than both lyophilized samples. In the context of dairy processing, whiteness is often associated with the light-scattering properties of the particles. Spray-dried powders consist of small, smooth, spherical particles that scatter light uniformly, creating a visual perception of high whiteness [58]. Additionally, the pH (7.5) of this sample likely contributed to this effect. At alkaline pH, the dissociation of casein micelles and the swelling of protein structures change the refractive index and scattering behavior of the particles, which have been shown to decrease turbidity in liquid systems and likely result in a lighter, whiter appearance once dried [60]. Lyophilized samples, while processed at lower temperatures, resulted in lower WI values (74–75). This is attributed to the larger, irregular, sheet-like morphology of the particles, which do not scatter light as effectively as spheres. Furthermore, the lack of alkaline-induced dissociation in these samples means that natural pigments remain in a more concentrated and visible form within the solid matrix [58].

Finally, the negative a* values across all samples indicate the presence of green tones, typical of the riboflavin (vitamin B2) content naturally present in whey. The lyophilized samples exhibit significantly more greenish (more negative a*) and yellowish (higher b*) tones than the spray-dried sample [61].

In addition, the significantly higher yellowness (b* ≈ 23) in samples BWH Lyophilized and BWH Lyophilized Prev. Crist-Filtr. is characteristic of lyophilization. Because this method avoids high heat, it preserves the integrity of heat-sensitive compounds and natural pigments. Conversely, the spray-dried sample shows a much lower b* value (7.28), indicating a loss of this characteristic yellowish tone. This could be due to the short-term exposure to high temperatures during atomization, which may have subtly altered the pigments, or, more likely, the high alkalinity, which can shift the absorption spectra of certain compounds or physically “dilute” them through the structural expansion of the protein matrix [62].

Based on the comparative evaluation, the spray-dried BWH was selected for subsequent analyses and studies, as it exhibited water activity values consistent with adequate product stability, together with more favorable physical and chromatic properties for its use as a powdered dairy ingredient. In addition, spray drying is the unit operation most widely applied at the industrial level for the processing of high whey volumes, which supports its selection in view of the future scalability and technology transfer of the process.

### 3.6. Peptide Profile of BWH by HPLC

**Figure 1:**
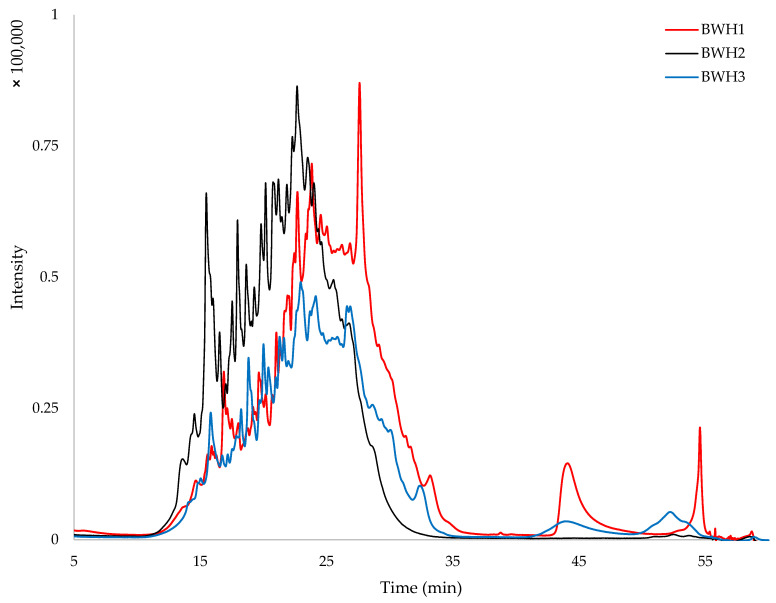
HPLC peptide profiles of bioactive whey hydrolysate (BWH) obtained from three different industrial whey batches.

The peptide profiles of the bioactive whey hydrolysate (BWH) obtained from different industrial whey batches were evaluated by reverse-phase HPLC (Figure 1). The chromatographic analysis showed comparable peptide profiles among the analyzed samples, with no evident qualitative differences in peak distribution or retention times. The peptides detected in all BWH samples eluted predominantly between 10 and 30 min, indicating a consistent peptide population across batches [63,64].

The elution range observed is characteristic of low- to medium-molecular-weight peptides with moderate hydrophobicity, which are commonly associated with bioactive properties, particularly angiotensin-converting enzyme (ACE) inhibitory activity. Several studies have reported that antihypertensive peptides derived from whey proteins elute within similar retention time windows under reverse-phase chromatographic conditions, due to their amino acid composition and hydrophobic character [63,65].

It should be noted, however, that retention time in reverse-phase HPLC is not an intrinsic property of peptides but is strongly influenced by chromatographic parameters such as column chemistry and dimensions, elution gradient, organic solvent composition, acidic modifier, flow rate, temperature, and the chromatographic system employed. Therefore, while the retention time window observed in this study is consistent with those reported for antihypertensive or ACE-inhibitory peptides under comparable RP-HPLC conditions, direct comparison of absolute retention times across different studies should be interpreted with caution.

The absence of marked differences among peptide profiles suggests that the enzymatic hydrolysis process was reproducible despite the use of whey obtained from different industrial batches. This consistency is relevant from both a technological and industrial perspective, as it indicates that the hydrolysis conditions applied are robust and capable of generating comparable peptide fractions from variable raw materials [66]. Such reproducibility is essential for the development of functional ingredients intended for large-scale production, where batch-to-batch variability of whey is unavoidable.

Overall, the HPLC peptide profiling confirms the generation of a stable and homogeneous peptide fraction enriched in peptides eluting within a retention time range commonly associated with antihypertensive activity, supporting the potential functional value of the BWH produced under the evaluated processing conditions [67,68].

### 3.7. Peptidomic Characterization of BWH

The peptidomic analysis of the three bioactive whey hydrolysate (BWH) samples by LC–MS/MS enabled an in-depth characterization of the peptide repertoire generated during enzymatic hydrolysis. As shown in Table 2, the total number of *de novo* peptides identified per sample ranged from 2126 to 3363 sequences, with an average of 2763 peptides across the three batches. These results confirm the high efficiency of the hydrolysis process in releasing a complex and diverse peptidome from whey proteins, in agreement with previous studies reporting extensive peptide generation under controlled enzymatic hydrolysis of dairy proteins [69,70]. The high peptide diversity observed can be attributed to the structural features of the major whey proteins, particularly β-lactoglobulin and α-lactalbumin, which present multiple protease-susceptible cleavage sites. Combined with the specificity of the enzymes employed, these characteristics favor the formation of partially overlapping peptides and contribute to the complexity of the peptidomic profile, as widely described for whey protein hydrolysates with functional potential [64,69,71].

The peptide size distribution is also relevant for the interpretation of ACE-inhibitory activity. Previous studies have reported that lower molecular weight peptide fractions tend to retain or concentrate ACE-inhibitory activity more effectively than higher molecular weight fractions, probably because shorter peptides can interact more efficiently with the ACE active site. In this context, the predominance of short- to medium-length peptides in BWH supports the observed ACE-inhibitory activity and agrees with recent evidence showing size-dependent differences in the bioactivity of protein hydrolysate fractions [72].

Despite the variability in the total number of *de novo* peptides among samples, the number of sequences identified as potentially antihypertensive remained relatively stable, ranging from 95 to 105 peptides. This observation suggests that, although the overall extent of hydrolysis may differ between batches, the release of peptides associated with angiotensin-converting enzyme (ACE) inhibitory activity is linked to conserved regions within whey proteins that are consistently cleaved during enzymatic processing, as previously reported for dairy-derived bioactive peptides [19,24,73]. The relatively low proportion of antihypertensive peptides compared to the total peptidome is consistent with literature indicating that only a limited fraction of peptides generated during protein hydrolysis exhibits specific bioactivities. Nevertheless, several studies have demonstrated that the antihypertensive effect of protein hydrolysates is often associated with the combined action of multiple peptides present in the matrix rather than with a single highly active sequence [69,74].

**Figure 2:**
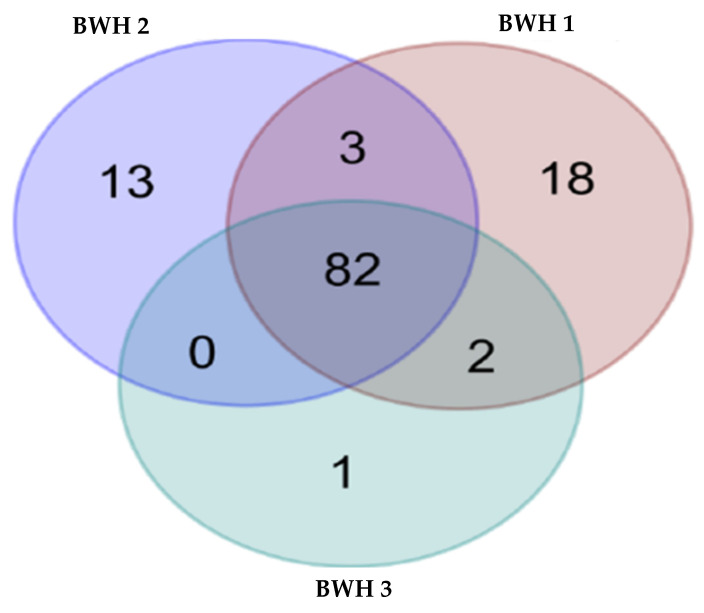
Venn diagram comparison of antihypertensive peptides identified in the three samples.

Overall, the Venn diagram (Figure 2) analysis highlights the presence of a conserved core of at least 82 antihypertensive peptides consistently identified across all analyzed samples, indicating a high level of reproducibility in the generation of bioactive sequences under the applied hydrolysis conditions. Similar behavior has been reported for dairy protein hydrolysates, where the release of angiotensin-converting enzyme (ACE) inhibitory peptides is primarily associated with the preferential cleavage of conserved regions within β-lactoglobulin and α-lactalbumin, which act as recurrent precursors of bioactive peptides [74,75]. The coexistence of a common bioactive peptide profile with some sample-specific peptides agrees with previous peptidomic studies. This finding supports the concept that the ACE-inhibitory activity of protein hydrolysates results from the combined action of multiple peptides rather than from a single dominant sequence [74,76,77].

### 3.8. ACE-Inhibitory Activity

All bioactive whey hydrolysate (BWH) samples obtained from different production batches exhibited high angiotensin-converting enzyme (ACE) inhibitory activity (Table 3). Mean inhibition values ranged from 89.8 ± 0.9% for BWH 1 to 97.0 ± 1.7% and 97.6 ± 0.5% for BWH 2 and BWH 3, respectively (mean ± SD; *n* = 3). One-way ANOVA revealed significant differences among batches (F (2,6) = 44.9, *p* < 0.001). Post hoc analysis (Tukey’s HSD, *p* < 0.05) indicated that BWH 2 and BWH 3 showed significantly higher ACE-inhibitory activity than BWH 1, while no significant differences were observed between BWH 2 and BWH 3. The low intra-batch variability observed confirms good analytical repeatability of the assay, consistent with the robustness reported for HHL-based ACE inhibition methods [40,78,79].

**Figure 3:**
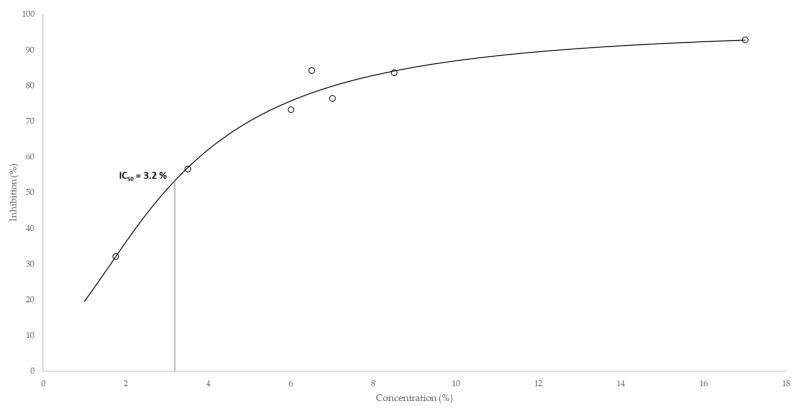
Dose–response curve of ACE-inhibitory activity of BWH used for IC_50_ estimation.

In addition to the fixed-concentration ACE-inhibitory assay, a dose–response analysis was performed to estimate the inhibitory potency of BWH. ACE inhibition increased with BWH concentration, ranging from 32.1% at 1.75% BWH to 92.9% at 17% BWH (Figure 3). Based on the fitted inhibition curve, the IC_50_ value was estimated as 3.20% BWH under the assay conditions. Captopril was used as a pharmacological ACE-inhibitory reference and confirmed the adequate responsiveness of the assay. Moreover, the low standard deviations observed among triplicate measurements and the consistent inhibitory activity across independent BWH production batches support the reliability and repeatability of the ACE-inhibitory activity determination.

The high inhibition levels observed across all samples are consistent with previous studies demonstrating that whey protein hydrolysates are rich sources of ACE-inhibitory peptides, generated through enzymatic hydrolysis of β-lactoglobulin and α-lactalbumin. These peptides often exhibit strong affinity for the ACE active site due to favorable structural features, including short peptide length and the presence of hydrophobic or aromatic residues at the C-terminal position, which enhance enzyme inhibitor interactions [63,80]. Recent peptide identification studies using LC–MS/MS and molecular docking have further confirmed that multiple whey-derived peptide sequences can contribute synergistically to overall ACE inhibition [73,79].

The significantly lower inhibition observed for BWH 1 compared to BWH 2 and BWH 3 suggests batch-to-batch variability in peptide composition, even when the hydrolysis process is performed under controlled conditions. Such variability has been widely reported for protein hydrolysates and is commonly attributed to differences in the relative abundance of bioactive peptide sequences, degree of hydrolysis, or minor variations in raw material composition. Similar batch-dependent effects on ACE-inhibitory activity have been described for whey hydrolysates produced by enzymatic or fermentation-assisted processes [24,81].

The absence of significant differences between BWH 2 and BWH 3, both exhibiting inhibition values close to 98%, suggests the occurrence of a plateau effect at the concentration tested. At high inhibitor concentrations, the ACE active sites may become largely saturated, limiting the sensitivity of percentage inhibition measurements to discriminate subtle differences among samples. This behavior has been previously described for HHL-based spectrophotometric assays and highlights a known limitation of single-point inhibition measurements when inhibition approaches maximal levels [82,83,84]. Under such conditions, complementary analysis using dose-response curves and IC_50_ determination is recommended to achieve finer discrimination of inhibitory potency [85].

Methodologically, the ACE inhibition assay employed in this study is one of the most widely used and validated approaches for screening the antihypertensive potential of food-derived peptides. However, it is well established that absolute inhibition values can be influenced by experimental parameters such as enzyme and substrate concentrations, incubation time, pH, and ionic strength. Consequently, careful reporting of assay conditions, as performed in the present study, is essential for inter-study comparability [40,82]. The low variability observed among triplicate measurements further supports the reliability of the results obtained under the selected conditions [63,86].

### 3.9. Stability of Bioactive Whey Hydrolysate Under Storage and Thermal Processing Conditions

**Figure 4:**
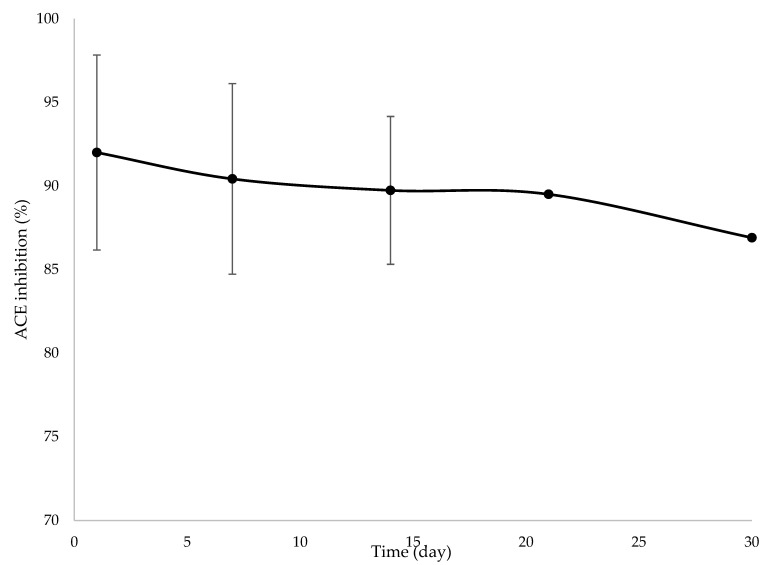
ACE-inhibitory activity of reconstituted bioactive whey hydrolysate (BWH, 17% *w*/*v*) during storage at room temperature over 30 days. Values are expressed as mean ± standard deviation (*n* = 3).

**Figure 5:**
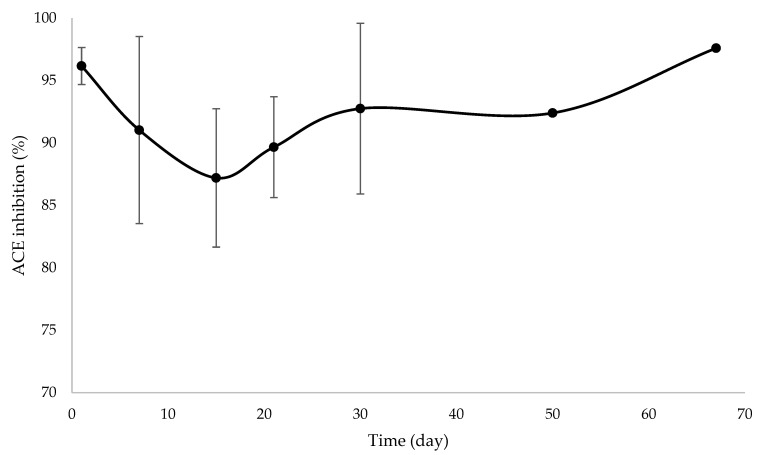
Record of the ACE-inhibitory activity exerted by BWH under refrigerated conditions over 68 days.

The results demonstrate that BWH retained high ACE inhibitory activity during storage at both room temperature and refrigerated conditions. As shown in Figure 4 and Figure 5, ACE inhibitory activity was preserved over the evaluated storage period without significant decreases, indicating a high functional stability of the ingredient under conditions relevant to functional food applications. This behavior is consistent with previous reports on protein hydrolysates, which indicate that low-molecular-weight peptides exhibit high stability during storage when maintained under moderate temperature and humidity conditions [87].

**Figure 6:**
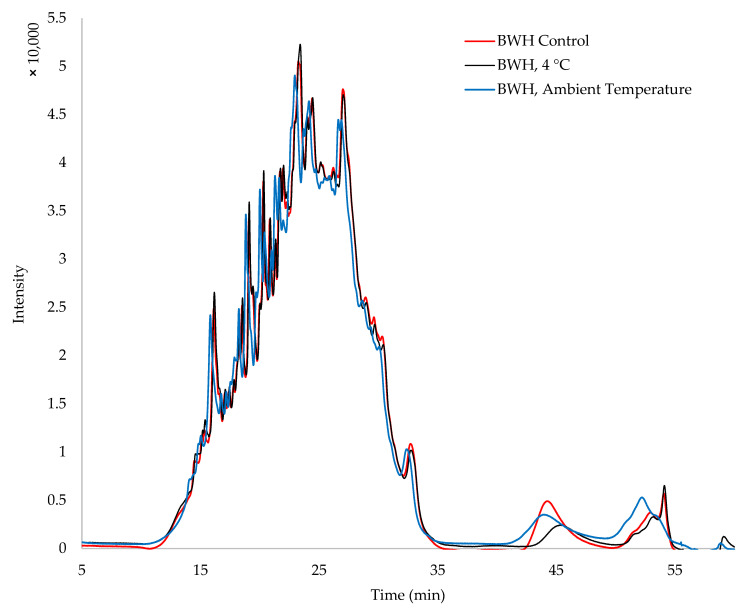
Peptide profile of BWH samples subjected to room temperature and refrigerated storage.

Peptide profiling by HPLC did not reveal relevant alterations following storage at either room temperature or refrigeration, as comparable chromatographic patterns were observed across conditions (Figure 6). The absence of noticeable changes in the chromatographic profiles suggests that no significant degradation, aggregation, or loss of major peptide fractions occurred during storage. This observation agrees with recent reviews on whey proteins and bioactive peptides, which indicate that the overall peptidome structure can remain stable during extended storage periods, particularly in systems lacking residual enzymatic activity [21].

Regarding thermal stability, BWH subjected to slow pasteurization conditions (62–64 °C for 30 min) did not exhibit significant differences in ACE inhibitory activity compared to untreated BWH, as summarized in Table 4. This result confirms that the bioactive peptides present in BWH are resistant to moderate thermal treatments, in agreement with previous studies reporting that the antihypertensive activity of peptides derived from dairy proteins is preserved after mild heating processes commonly applied in the food industry [88,89].

**Figure 7:**
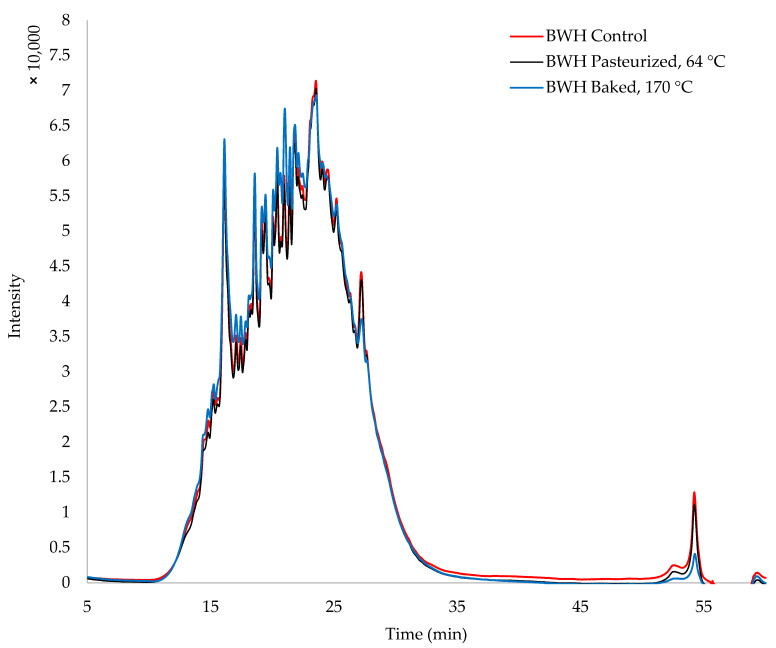
Peptide profile of BWH samples exposed to pasteurization (64 °C) and baking (170 °C) temperatures.

In contrast, exposure of BWH to 170 °C for 30 min, simulating baking conditions, resulted in a significant reduction in ACE inhibitory activity, reaching values close to 70% (Table 4). This decrease can be attributed to heat-induced reactions, such as thermal modifications and Maillard-type reactions, which may affect amino acid residues critical for ACE inhibition. Recent studies have shown that glycation and other heat-related modifications can negatively modulate the bioactivity of peptides, even in the absence of extensive peptide degradation [90]. Despite the reduction in bioactivity observed after the high-temperature treatment, the HPLC peptide profile did not show major changes in the overall chromatographic distribution (Figure 7). This suggests that the loss of ACE inhibitory activity was not associated with extensive peptide breakdown, but rather with subtle chemical modifications affecting the functionality of specific peptide sequences responsible for enzyme inhibition. Similar observations have been reported in the literature, where minor structural alterations can significantly impact bioactivity, while the global chromatographic fingerprint remains largely unchanged [86,89,91,92].

From a technological perspective, these results indicate that BWH exhibits high functional and structural stability under typical storage conditions and moderate thermal processing, supporting its potential application as a functional ingredient in refrigerated, liquid, or semi-solid food products. However, the partial loss of activity observed under baking conditions suggests that additional formulation strategies, such as peptide encapsulation, may be required to preserve bioactivity in products subjected to intense thermal processing. This approach has been widely proposed in the recent literature as an effective strategy to enhance the thermal stability and functional performance of bioactive peptides in complex food matrices [93,94].

### 3.10. Incorporation of Bioactive Whey Hydrolysate into Yogurt and Orange Juice

#### 3.10.1. Yogurt

**Figure 8:**
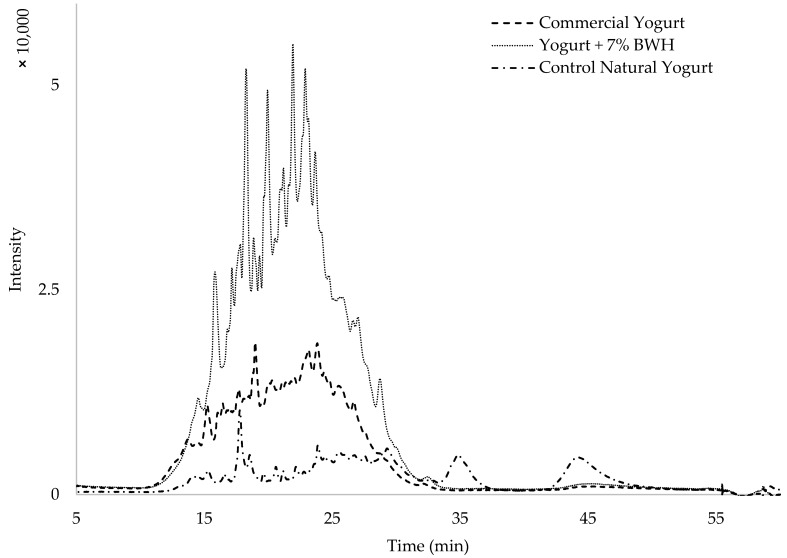
Antihypertensive peptide profile in yogurt samples.

ACE-inhibitory activity differed significantly among yogurt formulations (Table 5). The laboratory control yogurt showed low inhibition (5.5 ± 0.7%), whereas commercial yogurt exhibited moderately higher values (15.6 ± 1.1%). These findings are consistent with reports for unfortified fermented dairy products, in which ACE inhibition generally remains below 20% and is mainly attributed to limited release of casein-derived peptides during lactic fermentation [95,96]. The higher inhibition observed in commercial yogurt agrees with evidence that industrial formulations with higher protein content, addition of milk solids, and starter cultures with greater proteolytic activity favor the generation of antihypertensive peptides [97]. Fortification with 7% BWH led to a marked increase in ACE inhibition (77.5 ± 0.2%; Table 5), comparable to values reported for whey protein hydrolysates evaluated in model systems (70–90%), depending on hydrolysis degree and protease specificity [98]. RP-HPLC profiles (Figure 8) revealed increased signal intensity and chromatographic complexity in BWH-fortified yogurt, indicating that the yogurt matrix did not substantially limit peptide functional availability. This is notable given that partial bioactivity losses due to protein–peptide interactions or gel entrapment have been reported in other fortified yogurt systems [99].

#### 3.10.2. Juice

ACE-inhibitory activity also differed among orange juice formulations (Table 6). Natural orange juice showed moderate inhibition (21.5 ± 1.2%), which cannot be attributed to peptides due to the low protein content of this matrix. Previous studies indicate that citrus phenolics and flavanones may partially inhibit ACE or interfere with in vitro assays, explaining the basal activity observed [100]. Addition of 7% BWH markedly increased ACE inhibition to 87.1 ± 0.3% (non-pasteurized) and 85.4 ± 0.4% (pasteurized) (Table 6), in agreement with reports describing high antihypertensive potential of whey hydrolysates in liquid matrices, where activity is driven by the peptide pool [79].

Nevertheless, the slightly lower ACE-inhibitory activity observed in pasteurized BWH-fortified juice compared with the BWH-alone stability assay may reflect matrix-dependent effects. Unlike reconstituted BWH, orange juice is an acidic matrix containing sugars, organic acids, and phenolic compounds, which may influence peptide availability, peptide–matrix interactions, or the in vitro ACE-inhibitory response after heating [101]. In addition, citrus phenolics and flavonoids may contribute to ACE inhibition or influence the assay response [102]. Heat-induced reactions, including Maillard-type modifications, may also affect the bioactivity of protein-derived peptides during processing [103]. Therefore, thermal stability should be interpreted as matrix-dependent, although the non-significant difference between pasteurized and non-pasteurized fortified juice indicates that BWH retained most of its ACE-inhibitory activity under the applied pasteurization conditions.

**Figure 9:**
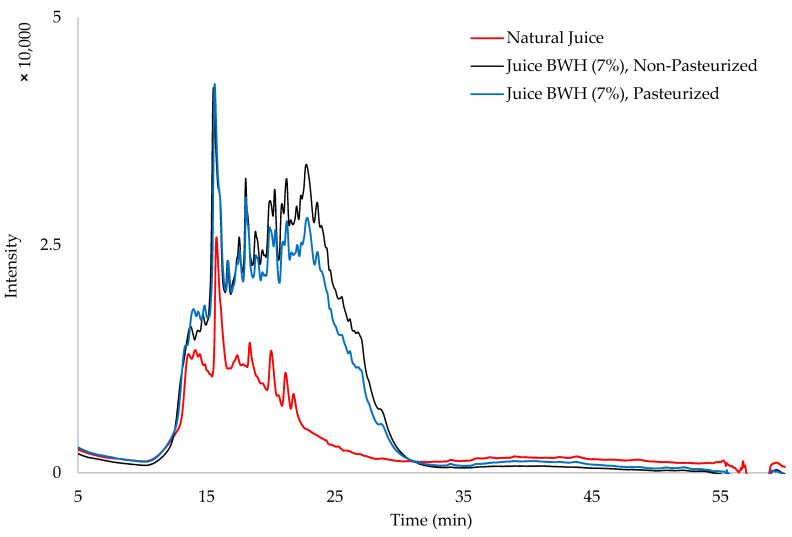
Antihypertensive peptide profile in juice samples.

The lack of relevant differences between pasteurized and non-pasteurized fortified juices indicates adequate thermal stability of the bioactive fraction under the applied conditions, consistent with the reported heat resistance of many whey-derived ACE-inhibitory peptides, particularly in acidic matrices [87]. RP-HPLC profiles (Figure 9) largely overlapped between pasteurized and non-pasteurized fortified juices, supporting the observed stability of ACE inhibition and suggesting minimal impact of thermal treatment on overall peptide composition.

Although the increase in ACE-inhibitory activity after BWH incorporation is strongly supported by the peptide profile generated through enzymatic hydrolysis, the contribution of matrix-derived and non-peptide components should also be considered. Basal ACE-inhibitory activity was observed in both control food matrices. In yogurt, this activity may be associated with peptides naturally released during lactic fermentation, whereas in orange juice it may be related to citrus phenolics or flavanones, or to possible interactions with the in vitro assay. In addition, BWH is a complex ingredient that may contain residual minerals, lactose-derived compounds, or Maillard reaction products formed during drying, which could partially influence the measured ACE-inhibitory response. Therefore, although the peptidomic results support an important contribution of BWH-derived peptides, the present study cannot fully exclude complementary effects from non-peptide compounds or from the food matrices themselves. Future studies should include non-hydrolyzed whey controls, peptide-depleted fractions, ultrafiltration-based peptide/non-peptide separation, mineral-matched controls, and isolated matrix controls to confirm the relative contribution of peptides to the observed bioactivity.

Overall, the concordance between increased ACE-inhibitory activity and RP-HPLC peptide profiles in both matrices supports the accepted concept that antihypertensive effects arise from the combined action of multiple peptides rather than a single sequence [79,81].

In this context, results for BWH incorporation into yogurt and orange juice are consistent with prior stability studies demonstrating preservation of ACE-inhibitory activity and peptide profiles under storage and processing. Nevertheless, a lower BWH incorporation level was used here compared with stability assays, which may explain differences in absolute ACE-inhibition values. Even at reduced levels, BWH retained high ACE-inhibitory activity in real food matrices, reinforcing its potential as a functional ingredient. Beyond this agreement with previous studies, the main contribution of the present work lies in the integrated validation of BWH, combining batch-to-batch reproducibility, peptide profiling, ACE-inhibitory potency, processing stability, and application in representative foods. This integrated approach provides a more complete technological and functional assessment than studies focused only on peptide identification or ACE inhibition in simplified systems [79].

## 4. Conclusions

This study demonstrated the potential of sweet whey as a valuable raw material for obtaining a bioactive whey hydrolysate (BWH) with ACE-inhibitory activity through controlled enzymatic hydrolysis using plant-derived proteases. The hydrolysate showed suitable physicochemical characteristics for use as a powdered ingredient, and peptide profiling by RP-HPLC and LC–MS/MS revealed a complex and reproducible peptide profile across independent production batches. The presence of conserved antihypertensive peptide sequences, together with the high ACE-inhibitory activity observed among batches, supports the functional consistency of the hydrolysis process.

BWH exhibited strong ACE-inhibitory activity, with low variability among replicate measurements and an estimated IC_50_ of 3.20% under the assay conditions. In addition, the hydrolysate maintained its ACE-inhibitory activity and peptide profile during storage at room temperature and under refrigeration, as well as after pasteurization-like treatment. A partial decrease in activity was observed after exposure to high-temperature treatment, suggesting that intense thermal processing may affect some bioactive peptide fractions. Preliminary incorporation trials in yogurt and orange juice showed that BWH retained ACE-inhibitory activity after addition to representative food matrices, supporting its potential use as a functional ingredient in dairy and beverage applications.

Overall, these findings highlight the integrated technological and functional validation of BWH, combining batch-to-batch reproducibility, peptide characterization, ACE-inhibitory potency, processing stability, and preliminary application in real food systems. This approach provides a broader assessment than studies focused only on peptide identification or ACE inhibition in model systems and supports the potential valorization of sweet whey into functional ingredients with ACE-inhibitory potential.

However, some limitations should be acknowledged. The incorporation trials in yogurt and orange juice were designed as preliminary food-matrix assays focused on functional retention, rather than as optimized final formulations. Therefore, the practical applicability of BWH-fortified products requires further evaluation through sensory analysis, complete physicochemical characterization, consumer acceptability studies, and dose optimization. In addition, future studies should address peptide bioaccessibility and bioavailability, long-term stability in formulated foods, and dedicated in vivo or clinical validation to further substantiate the health relevance of BWH-based functional foods.

## Figures and Tables

**Table 1:** Physicochemical property values for Bioactive Whey Hydrolysate (BWH) samples subjected to different drying treatments.

Sample	pH Value (-)	Moisture Content (%)	Apparent Density (g/mL)	Water Activity (-)	Chromatic Parameter			Whiteness Index
					L*	a*	b*	
BWH Lyophilized	6.70 ± 0.30 ^a^	11.07 ± 0.25 ^a^	0.59 ± 0.07 ^a^	0.36 ± 0.08 ^a^	90.23 ± 0.71 ^a^	−4.67 ± 0.10 ^a^	23.18 ± 0.47 ^a^	74.41 ^a^
BWH Spray Dried	7.50 ± 0.20 ^b^	11.15 ± 0.01 ^a^	0.62 ± 0.03 ^a^	0.29 ± 0.02 ^a^	97.96 ± 0.62 ^b^	−1.98 ± 0.33 ^b^	7.28 ± 1.27 ^b^	92.18 ^b^
BWH Lyophilized, previously crystallized and filtered	6.80 ± 0.60 ^a^	6.85 ± 0.36 ^b^	0.31 ± 0.03 ^b^	0.33 ± 0.05 ^a^	91.78 ± 0.89 ^a^	−4.01 ± 0.13 ^a^	22.45 ± 1.21 ^a^	75.74 ^a^

Values expressed as means ± standard deviation. Different lowercase (^a, b^) letters in the same column indicate significant differences (*p* ≤ 0.05) among BWH samples.

**Table 2:** Peptides identified in the different hydrolyzed samples.

Sample	*De Novo* Peptides	Antihypertensive Peptides
BWH 1	2802	105
BWH 2	3363	98
BWH 3	2126	95

**Table 3:** ACE-inhibitory activity of BWH samples.

Sample	ACE Inhibitory Activity (%)
BWH 1	89.8 ± 0.9 ^a^
BWH 2	97.0 ± 1.7 ^b^
BWH 3	97.6 ± 0.5 ^b^

Values are expressed as mean ± SD (*n* = 3). Different superscript letters indicate significant differences among samples according to Tukey’s HSD test (*p* < 0.05).

**Table 4:** Evaluation of the ACE-inhibitory activity of BWH before and after heat treatments.

Sample	ACE Inhibitory Activity (%)
BWH control	97.8 ± 0.6 ^a^
BWH pasteurized (64 °C)	97.1 ± 0.5 ^a^
BWH baked (170°)	70.3 ± 1.1 ^b^

Values are expressed as mean ± SD (*n* = 3). Different superscript letters indicate significant differences among samples according to Tukey’s HSD test (*p* < 0.05).

**Table 5:** Evaluation of BWH activity as an ingredient in yogurt.

Sample	ACE Inhibitory Activity (%)
Control natural yogurt	5.5 ± 0.7 ^a^
Comercial yogurt	15.6 ±1.1 ^b^
Yogurt + 7% BWH	77.5 ± 0.2 ^c^

Values are expressed as mean ± SD (*n* = 3). Different superscript letters indicate significant differences among samples according to Tukey’s HSD test (*p* < 0.05).

**Table 6:** Evaluation of BWH activity as an ingredient in juice.

Sample	ACE Inhibitory Activity (%)
Natural juice	21.5 ± 1.2 ^a^
Juice BWH (7%) unpasteurized	87.1 ± 0.3 ^b^
Juice BWH (7%) pasteurized	85.4 ± 0.4 ^b^

Values are expressed as mean ± SD (*n* = 3). Different superscript letters indicate significant differences among samples according to Tukey’s HSD test (*p* < 0.05).

## Data Availability

The data presented in this study are available on request from the corresponding author.

## Abbreviations

The following abbreviations are used in this manuscript:

ACE	Angiotensin-Converting Enzyme
BWH	Bioactive Whey Hydrolysate
